# Patient characteristics associated with their level of twelve-step attendance prior to entry into treatment for substance use disorders

**DOI:** 10.1186/s13722-025-00542-5

**Published:** 2025-02-14

**Authors:** Marc Galanter, William L. White, Michael L. Dennis, Brooke Hunter, Lora Passetti, Dan Lustig

**Affiliations:** 1 Department of Psychiatry, New York University School of Medicine, 462 First Avenue, Twentieth Floor, New York, NY 10016 USA; 2 Lighthouse Institute, Chestnut Health Systems, Punta Gorda, FL USA; 3 Lighthouse Institute, Chestnut Health Systems, Chicago, IL USA; 4 Chestnut Health Systems, Normal, IL USA; 5 Haymarket Center, Chicago, IL USA

**Keywords:** Alcoholics Anonymous, Narcotics Anonymous, Characteristics prior to treatment

## Abstract

**Background:**

The availability of the fellowships of Alcoholics Anonymous and Narcotics Anonymous in community settings is extensive and patients admitted to treatment programs for substance use disorder may therefore have previously attended meetings of these two Twelve Step (TS) programs. Data on such prior attendance and related clinical findings, however, are not typically available. They can, however, be relevant to how ensuing treatment is planned. We therefore undertook this study to ascertain the feasibility of evaluating how the level of TS attendance prior to treatment entry can be evaluated, and to determine clinically relevant findings that are associated with such attendance.

**Methods:**

Over the course of 2022, 3,125 patients were admitted to a large urban multimodal United States-based treatment center. All patients were administered the structured interview-based Global Appraisal of Individual Needs upon admission. This instrument is employed to evaluate substance use, demographics, and related psychosocial variables. Clinically related variables were analyzed relative to whether given respondents have a history of any TS group attendance prior to admission.

**Results:**

Distinctions were found between the 57.3% of respondents who had previously attended any TS meetings and the 42.6% who had not attended any meetings. Compared to respondents who had never attended TS meetings, those who had ever attended scored higher on emotional problems (*p* <.001, d = -0.58), and had more likely undergone previous SUD treatment (*p* <.001, d = 0.80). They were less likely to use substances in unsafe situations (*p* <.001, d = -0.55) and were less likely to express reluctance to remain abstinent (*p* <.001, d = -0.50). The 11% of respondents who considered themselves regular TS members reported a lower frequency of recent substance use (*p* <.001, d = -0.80) and were more likely to have attended intensive outpatient (*p* <.001, 0.46) and residential (*p* <.001, 0.44) treatment than patients who did not consider themselves regular attenders.

**Conclusions:**

Examination of TS attendance prior to treatment admission is feasible. Findings can be clinically relevant for differential treatment planning and can also serve as a basis for further research into the role of TS participation in community settings.

## Background

There is a diversity of modalities that can be undertaken for people who apply for substance use disorder (SUD) treatment in community settings. Because of this diversity, there is value in planning treatment relative to their history of involvement with different types of services. We elected to study how one aspect of applicants’ prior experience, namely attendance at Twelve Step (TS) meetings, can be evaluated for use clinically at the time of entry into treatment. Exposure to this experience is quite common among treatment applicants, as a probability sampling of the US population revealed that 5.9% of the overall population indicated that they had received “treatment” in a self-help group, typically Alcoholics Anonymous or Narcotics Anonymous [1].

Most determinants of the outcome of SUD treatment relative to TS experience have been studied both during treatment and after discharge. This is illustrated by attendance at TS meetings, as follows: for patients who received residential care [2], in both public and private treatment programs [3], in relation to group-based TS facilitation [4], in outcome for adolescent outpatients [5], in acquisition of a sponsor [6, 7], in association with group cohesion during treatment [8], and in a therapeutic alliance between sponsor and sponsee [9].

TS attendance before treatment entry, however, can be relevant in planning treatment suitable for given patients. For example, providers may tailor clinical approaches based on the level of prior involvement in and knowledge of TS programs. We undertook this study to ascertain how an assessment of such attendance can be carried out. We also chose to examine patient characteristics related to prior TS experience. TS experience is common, and findings on its role before treatment entry have not been reported. We therefore undertook this study to answer two questions:

1. How can persons entering a community-based SUD treatment program be characterized, by means of a formalized interview format, based on whether or not they have had prior TS experience? This can, for example, be ascertained by structured interviews at the time of admission, including experience with prior TS experience.

2. What differences are there between applicants coming for treatment who self-designate as (a) currently active TS members, (b) those who had previously attended TS groups, and (c) those who have never attended TS groups?

## Method

### Sample

The current study is a retrospective cohort study, which was carried out on intake data provided by all persons with a SUD presenting for treatment at the Haymarket Center in the United States, in Chicago, IL, during the calendar year 2022. To be included in the analysis, individuals had to complete the Global Appraisal of Individual Needs (GAIN) [10] intake assessment (*N* = 3190).

Patients admitted to the Haymarket Center treatment facilities are interviewed employing the GAIN survey, and are included in this study. A small number, however, such as those cognitively compromised, are psychotic, or cannot reply to the items in the survey, and are excluded. They had to provide a valid response (“yes” or “no”) to items such as: “Do you regularly attend AA or NA meetings?” or “Have you ever attended TS or self-help meetings?” Sixty-five individuals were excluded from the analysis due to missing data on these two key items resulting in a final sample of 3,125. Respondents were subsequently divided into cohorts based on responses to these items: “Regular TS Attenders” versus “Non-Regular TS Attenders” and “Ever TS Attenders” versus “Never TS Attenders.”

### The Haymarket Center

This Center was established in 1975 and is a large not-for-profit community-based treatment facility for SUD, serving 12,000 people annually, and providing detoxification, residential, and outpatient treatment to a diverse population of low-income patients regardless of ability to pay [11]. At the time of data collection, some persons entering treatment were provided opioid maintenance, but only methadone was provided in this capacity at that time.

### The Global Appraisal of Individual Needs (GAIN)

In order to address the questions raised, an instrument was chosen to be employed through structured interview that addresses the specifics of both community and professional issues relevant to the TS experience of treatment applicants. The GAIN was developed as a means of assessing persons admitted for SUD treatment for demographic, behavioral, and diagnostic issues. The full GAIN is administered as a series of interview items designed to address research and clinical program needs [12, 13] and is conducted over a period of three hours. Training for GAIN interviewers is carried out over a one-week structured course. The GAIN has been applied in research initiatives such as a Rasch analysis of its items on its Substance Problem Scale, validation of its Self Help Involvement Scale [14], a determination of a continuum of SUD severity [15], treatment planning [10], specific clinical issue areas related to treatment outcome [16], and adaptation internationally [17]. Scales and indices obtained from the GAIN and analyzed in the current study are described in Table 1. Additionally, individual items from the GAIN that provide additional insight into various domains of life (i.e., mental health, vocational activities, legal system involvement, substance use, and prior treatment) were analyzed. Only data collected at intake to treatment was analyzed; no follow-up data were available. The GAIN consists of the following sections: social background on substance use, substance frequency scale, social background, physical and mental health, and risk behavior. Interviewers are rehearsed for how to apply the items to patients and are certified for their competency in accordance with a structural manual.

**Table 1:** Description of scales and indices included in study

Scale/Index Name	Abbreviation	Definition	Interpretation
Emotional Problem Scale	EPS α = 0.81	Average (expressed as a percent) of items for the recency and days (during the past 90): bothered by or kept from responsibilities because of emotional problems, disturbed by memories, and having problems paying attention or with self-control	Higher values indicate greater emotional problems. Values range from 0-100, with values greater than 14 indicating high severity of issues that should be taken into consideration in treatment planning.
Mental Health Treatment Index	MHTI	Percentage of days in the past 90 in which a client received mental health treatment, including days on medication	Higher scores indicate more involvement in mental health treatment in the past 90 days
Substance Problem Scale	SPS α = 0.83	The average number of past month symptoms of substance use disorders and substance induced social, health and psychological disorders based on the DSM-5.	Higher scores on this scale represent greater severity of drug problems. The scale includes physiological, psychological and social criteria, as well as an item on comorbid use with drugs that is likely to exacerbate the other problems
Substance Frequency Scale	SFS α = 0.72	The average percentage of days out of the past 90 reporting alcohol or other drug (AOD) use, heavy AOD use, and problems from AOD use.	Higher scores represent increasing frequency of substance use, days staying high most of the day, and days causing problems. People with scores over 0.14 may have considerable difficulty stopping without significant assistance.
Treatment Motivation Index	TMI	Count of items endorsed regarding the client’s perception of sources of external pressure to be in treatment and their own need for treatment, support for treatment, and hope for help through treatment.	Higher scores on this scale suggest more motivation for the individual to be in treatment.
Self-Help Involvement Scale	SHIS α = 0.91	Indicates level of involvement and participation in self-help activities.	A higher score indicates more involvement.

*Notes.* Cronbach’s α was calculated to provide a measure of internal consistency for all scales

This project was approved by the Institutional Review Board of Chestnut Health Systems. The survey data were anonymized without items that would allow for obtaining respondents’ respective identities. The datasets used and/or analyzed during the current study are available from the corresponding author on reasonable request.

### Analysis

Two separate cohorts were analyzed in this study: (1) “Regular TS Attenders” versus “Non-Regular TS Attenders”, and (2) “Ever TS Attenders” versus “Never TS Attenders”. Characteristics for each cohort were compared and analyzed using chi-square statistics for categorical or binary outcomes, and *t*-tests were used for continuous outcomes. Furthermore, Cohen’s *d* effect sizes were calculated for continuous metrics, while Cohen’s *h* was calculated for binary comparisons [18]. Cohen’s *d* and *h* values can be interpreted as follows: values ranging from 0.20 to 0.49 are considered small effects sizes, values ranging from 0.50 to 0.79 are considered medium effect sizes, and values equal to or greater than 0.80 are considered large effect sizes. In order to account for multiple comparisons, results reported in this study were limited to those where the *p*-value was less than 0.001 and Cohen’s *d* or *h* values were 0.20 or greater.

Rates of missing data were minimal across most items and were handled by listwise deletion. The valid sample size for each analyzed outcome is reported in Tables 2 and 3. Analyses were conducted using SPSS Statistics Version 29.0.2.

**Table 2:** Comparison of regular 12-Step attenders versus non-regular TS attenders on characteristics across several domains assessed at intake to SUD treatment

	Regular			Non-Regular					
	N	Mean/%	SD/Count	N	Mean/%	SD/Count	*t/*χ^2^	*p*	Cohen’s *d/h*
***Demographics***									
Race									
African American	342	43.3%	148	2759	56.4%	1556	21.17	< 0.001	-0.26
Hispanic	342	12.0%	41	2762	13.0%	359	0.28	0.594	-0.03
White	342	41.5%	142	2760	27.1%	748	30.87	< 0.001	0.30
Mixed	333	1.8%	6	2824	1.7%	48	0.00	0.984	0.01
Other	333	1.5%	5	2824	1.7%	48	0.14	0.709	-0.02
Female	345	32.2%	111	2778	33.7%	936	0.32	0.573	-0.03
Age	345	42.6	11.75	2778	43.7	12.45	-1.53	0.127	-0.09
Ever been homeless	343	74.1%	254	2768	67.6%	1872	5.82	0.016	0.14
***Mental Health***									
Emotional Problems Scale	345	32.27	24.09	2688	25.70	22.90	5.00	< 0.001	0.29
Days bothered by psychological problems	345	41.34	38.22	2688	32.83	36.88	3.91	< 0.001	0.23
Days disturbed by memories	345	24.45	34.23	2688	17.69	30.27	3.49	< 0.001	0.22
Mental Health Treatment Index	341	37.54	46.23	2772	23.61	40.68	5.31	< 0.001	0.34
***Vocation***									
Employed within past year	341	52.8%	180	2760	42.0%	1159	14.50	< 0.001	0.22
***Legal***									
Current criminal justice system involvement	339	36.9%	125	2675	27.4%	733	13.29	< 0.001	0.20
***Substance Use***									
Treatment Motivation Index	339	3.14	0.88	2685	2.91	1.10	4.35	< 0.001	0.21
Proportion of days using AOD in the community	345	0.54	0.41	2777	0.79	0.33	-11.11	< 0.001	-0.77
Substance Frequency Scale	345	24.53	20.15	2780	40.77	20.25	-14.06	< 0.001	-0.80
Substance Problem Scale	345	9.52	5.77	2776	11.39	4.60	-5.78	< 0.001	-0.41
Used when it was not safe	345	24.4%	84	2776	39.6%	1098	30.00	< 0.001	-0.33
Caused problems with other people	345	61.2%	211	2776	73.3%	2032	22.21	< 0.001	-0.26
Needed more to get high	345	50.1%	173	2776	73.7%	2047	83.19	< 0.001	-0.49
Experienced withdrawal	345	55.4%	191	2776	72.7%	2018	44.57	< 0.001	-0.36
Used more than meant to	345	67.5%	233	2776	83.3%	2311	50.52	< 0.001	-0.37
Unable to stop or cut down	345	71.0%	245	2776	85.9%	2385	51.39	< 0.001	-0.37
Spent a lot of time getting AOD	345	58.6%	202	2776	79.8%	2213	79.37	< 0.001	-0.47
***Prior Treatment***									
Any prior treatment?	345	92.8%	320	2776	73.6%	2043	61.25	< 0.001	0.54
Prior treatment modality:									
Outpatient	345	19.1%	66	2765	8.1%	224	44.7	< 0.001	0.33
Methadone Maintenance	345	13.3%	46	2780	13.3%	371	0	0.995	0.00
Intensive outpatient	345	33.9%	117	550	14.7%	81	80.83	< 0.001	0.46
Residential	345	83.5%	288	2781	64.4%	1791	50.04	< 0.001	0.44
Other	345	14.8%	51	2780	7.9%	220	18.28	< 0.001	0.22
How reluctant are you to remain abstinent?	282	2.64	10.82	1943	10.31	20.64	-9.63	< 0.001	-0.37
How reluctant are you to stop using AOD?	61	3.77	14.62	858	11.78	28.28	-3.8	< 0.001	-0.28

*Notes.* Alcohol and Other Drugs (AOD); Substance Use Disorder (SUD). This table presents results comparing regular versus non-regular Twelve-Step (TS) attenders on several characteristics measured at intake to SUD treatment. Categorical variables were analyzed using chi-square statistics, and continuous metrics were analyzed using a *t*-test. Cohen’s *h* was used to calculate the effect size for variables presented here as percentages, and Cohen’s *d* was used to calculate the effect size for variables presented here as means and standard deviations. Regular TS attenders are presented here as the comparator group, while non-regular TS attenders were treated as the referent group. Thus, positive effect sizes indicated the regular TS attenders had a greater mean or percentage for the item, while negative values indicate that non-regular TS attenders had a greater mean or percentage for the item

**Table 3:** Comparison of individuals presenting to SUD treatment who had ever attended TS versus those who had never attended TS based on characteristics assessed at intake to SUD treatment

	Ever 12-Step (*n* = 1,790)		Never 12-Step (*n* = 1,331)				
	Mean/%	SD/Count	Mean/%	SD/Count	*t/*χ^2^	*p*	Cohen’s *d/h*
***Demographics***							
Race							
African American	51.2%	909	60.0%	792	23.55	< 0.001	-0.18
Hispanic	12.2%	216	13.9%	184	2.10	0.147	-0.05
White	33.4%	593	22.4%	296	44.65	< 0.001	0.25
Mixed	1.5%	26	2.1%	28	1.90	0.168	-0.05
Other	1.8%	32	1.6%	21	0.20	0.653	0.02
Ever been homeless	75.3%	1342	59.2%	784	91.62	< 0.001	0.35
***Mental Health***							
Emotional Problems Scale	30.77	24.07	20.65	20.39	12.67	< 0.001	0.50
Mental Health Treatment Index	29.12	43.56	19.95	38.16	6.15	< 0.001	0.24
***Substance Use***							
Treatment Motivation Index	3.14	1.01	2.67	1.11	12.19	< 0.001	0.43
AOD kept you from meeting responsibilities	62.6%	1119	75.7%	1005	60.41	< 0.001	-0.29
AOD use created unsafe situations	26.5%	474	53.2%	706	230.51	< 0.001	-0.55
AOD use caused you to give up important activities	57.2%	1022	70.6%	938	58.27	< 0.001	-0.28
***Prior Treatment***							
Any prior treatment?	90.1%	1612	56.4%	749	469.72	< 0.001	0.80
Prior treatment modality:							
Outpatient	12.2%	218	5.4%	72	41.50	< 0.001	0.24
Methadone Maintenance	16.1%	288	9.7%	129	26.99	< 0.001	0.19
Intensive outpatient	22.5%	402	9.2%	123	95.31	< 0.001	0.37
Residential	81.6%	1461	46.3%	616	428.25	< 0.001	0.76
Other	10.2%	182	6.6%	88	12.22	< 0.001	0.13
Currently in treatment for AOD	12.3%	220	5.0%	67	48.038	< 0.001	0.27
How reluctant are you to remain abstinent?	2.63	10.25	16.88	24.70	-17.39	< 0.001	-0.58
How reluctant are you to stop using AOD?	5.00	16.99	24.16	37.84	-8.13	< 0.001	-0.51

*Notes.* Alcohol and Other Drugs (AOD); Substance Use Disorder (SUD). This table presents results comparing individuals who had ever attended Twelve-Step (TS) versus those who had never attended TS on several characteristics measured at intake to SUD treatment. Categorical variables were analyzed using chi-square statistics, and continuous metrics were analyzed using a *t*-test. Cohen’s *h* was used to calculate the effect size for variables presented here as percentages, and Cohen’s *d* was used to calculate the effect size for variables presented here as means and standard deviations. Individuals who had ever attended TS are presented here as the comparator group, while those who had never attended TS were treated as the referent group. Thus, positive effect sizes indicated the “ever TS attenders” had a greater mean or percentage for the item, while negative values indicate that “never TS attenders” had a greater mean or percentage for the item

## Results

In 2022, 3,125 persons were evaluated employing the GAIN instrument upon admission to the Haymarket Center. Persons admitted were 67% male, with a mean age of 46.3 (SD 12.4). Racial self-designations were 53.6% African-American, 29.7% White, 12.6% Hispanic, and 3.4% other ethnicities. The minority (45.3%) of those admitted were employed at some time during the previous year, and 69.1% had experienced current or past homelessness. Of the entire sample, 75.6% had previously undergone treatment for multiple substances of misuse.

### Regular TS attenders

Of the respondents, 11% (*N* = 345) designated themselves as regular TS members. As indicated in Table 2 compared to other respondents, the regular TS attenders scored higher on the Emotional Problems Scale (EPS) and on the Mental Health Treatment Index (MHTI). They were more likely to have had some employment in the previous year. They scored lower on the Substance Problem (SPS) and Substance Frequency scales (SFS) and were more likely to have had prior treatment for an SUD than non-attenders (h = 0.54). There was no significant difference across the two groups on their respective principal drugs of misuse or in achieving a high school diploma.

As in Table 1, the GAIN included items constituting scores on the EPS. The regular TS attenders’ scores were driven by several component items of the EPS. They reported more days out of the past 90 days of being bothered by any nerve, mental, or psychological problems (d = 0.23), and more days out of the past 90 days of being disturbed by memories of things from the past that they did, saw, or had happened to them (d = 0.22).

### Respondents who are not TS attenders

On the SPS, responders who were not regular TS attenders (*N* = 2780) had more severe substance use problems, with the following items yielding the biggest differences between them and the regular attenders. They repeatedly used alcohol or other drugs (AOD) when it made their situation unsafe (h = -0.33), kept using AOD even though it caused problems with other people (h = -0.26), needed more AOD to achieve the same high (h =-0.49), experienced withdrawal (h = -0.36), used AOD in larger amounts than they meant to (h =-0.37), were unable to stop or cut down (h = -0.37), and spent a lot of time getting AOD, using AOD, or being high (h = -0.47). The following items were omitted from Table 2 due to non-significant difference between the two groups: gender, age, and ever been homeless.

### Those who had ever attended TS meetings

Those who reported having ever attended TS meetings (*N* = 1790) predominated over those who never attended (*N* = 1331), as seen in Table 3. Ever attenders had higher scores on the EPS, more prior mental health treatment, less reluctance for abstinence, and more prior substance use disorder treatment than those who never had TS experience. The two groups did not differ significantly in gender, age, achievement of a high school diploma, or scores on the SPS or SFS nor did they differ significantly in the substance employed.

Analysis of individual EPS items indicated that, out of the past 90 days, *ever* attenders were more likely to be bothered by any nerve, mental, or psychological problems (d = 0.42), were more likely to be kept from their responsibilities at work, home, or school by psychological problems (d = 0.30), and were disturbed by memories of things from the past that they did, saw, or had happen to them (d = 0.38). On the SPS, never attenders had more severe substance use problems in general, with the following items yielding the biggest differences between the two groups: AOD kept them from meeting responsibilities at work, school, or home (h = -0.29), they repeatedly used AOD when it made the situation unsafe (h = − 0.55) and caused them to give up important activities at work, school, or home (h = -0.28). The following items were omitted from Table 3 due to non-significance: gender, age, past year employment, current criminal justice system involvement, SFS, SPS, and the proportion of days using AOD out of the past 90 days. Both regular or ever attenders who attended TS meetings had less reluctance to accept abstinence as an option. A small portion of both groups had experience with methadone maintenance.

The substances that were most used in the previous 90 days were not significantly different across these groups: regular TS attenders, those who ever attended TS groups, and those with no prior TS experience.

## Discussion

Only 24% of people in the United States in 2022 who were in need of formal SUD treatment received such treatment [19]. Furthermore, treatment readmission rates are major contributors to the related SUD disease burden [20]. TS groups, however, are widely available and free of charge. They can play a role in addressing this deficit. Alcoholics Anonymous reports 1,350,415 members in the US [21] and Narcotics Anonymous reports 23,511 groups in the US [22]. It is therefore useful to consider the large portion of persons who have accessed such non-professional support, that is, by persons in the community who are not compensated for their assistance, such as fellow TS members, or members of a house of worship. While TS involvement during treatment may be examined in outcome studies, TS experience prior to treatment entry, even though likely common, is not typically assessed, in part because it operates largely independent of professional care. This study was therefore designed to examine clinical characteristics of the persons entering treatment in a large community-based program who did have prior experience with TS fellowships, to clarify their role in treatment entry.

Clinical assessment of patients’ status at the time of application and acceptance for entry into a clinical program can, however, be useful for choosing options for treatment. One example of this is the American Society of Addiction Medicine (ASAM) criteria for severity of SUD which allows for grading the level of treatment intensity appropriate relative to the severity of the illness [23]. The ASAM criteria are widely used by clinicians in evaluating patients for treatment and are therefore relevant to the findings reported here. Implementation of these criteria has been found to serve as predictive of patient retention in treatment, and it has also been used to estimate the extent of treatment available within a given population relative to SUD treatment available [24, 25].

Use of the GAIN in this study, however, has certain advantages. It offers a structured accounting of diverse aspects of the interviewee’s background, their access to care, substance use, health and mental health, and social adaptation. Additionally, it was structured and developed for applicability in clinical research and was previously employed in a number of empirical studies, and interviewers undergo extended structured training for certification for its use.

The GAIN has been used to evaluate treatment options as diverse as mindfulness training [26], assessment of potential suicidal behavior [27], and the potential for abstinence outcome in specific settings such as drug courts [28] and outcome-relative choice of residential or outpatient treatment [15]. It has also been employed in translation [29]. It has also been found to be in agreement with clinician evaluations for treatment planning based on the American Psychiatric Association diagnostic criteria and ASAMguidelines [10].

The relationship between TS involvement and clinical outcome is important, as it can bear on how the TS fellowships’ role is understood. It is typically evaluated *after* treatment, as in intensive outpatient [30] and inpatient settings [31], in long-term follow-up [32], and often with a meta-analysis of its use relative to other psychotherapeutic treatments [33]. Comparisons have also been made for special populations such as youth [31, 34] and for persons treated with pharmacotherapy for opioid use disorder [35]. 

We found that the large majority of applicants for treatment at the Haymarket Center (75.6%) had undergone previous treatment, illustrating that prior treatment can play an important role for some patients in characterizing issues and can be important in the response of persons to the modalities applied. Community-based SUD treatment programs vary in the psychosocial modalities offered by their respective staffs, but this is usually done with limited focus on the enrollees’ prior treatment experience. Because of the high prevalence of TS availability in most communities, we chose to employ the GAIN format to assess TS experiences *prior* to program entry. This was carried out by employing findings from the Haymarket Center to illustrate how the role of *prior* TS experience can be evaluated. It is worth noting, however, that use of this instrument can also be a basis for considering less common antecedents of prior treatment experience. For example, 13% of admitted patients have prior experience with methadone maintenance, reflecting an issue worthy of further investigation.

### TS attendance before treatment entry

Those who were regular TS attenders were more likely to be White and less likely to be Black than those who were not regular TS attenders, but among the ever attenders, this difference was significant only for Whites. Regarding persons beginning treatment, however, certain clinical issues do make clear that patient characteristics are associated with prior experience with TS attendance. Regular TS attenders before treatment entry were associated with a lower number of days using substances prior to intake than the other applicants admitted. Those who ever attended TS, however, were not significantly different in drugs used from those who never attended TS. This suggests that persons who were regular attenders upon applying for treatment may have a lower threshold of AOD use that motivated them to seek treatment than non-regular and never attenders. They may have also been encouraged by a sponsor or by other members to seek treatment.

The statistical analysis in the Results section as reported in the Tables merits review, as this can provide further clarity on the differential nature of access across the program’s applicant population. It can also illustrate issues that can be addressed in further research. The regular TS attenders also reported fewer drug-related problems than those who were not TS attenders. Also, those who ever attended TS groups were no different on drug problems from those who never went to TS. Regular TS attenders who were admitted to Haymarket are also more likely to be committed to abstinence. Both regular and ever TS attenders reported lower resistance to attend treatment and were less likely to be reluctant to stop using AOD and remain abstinent. Although never attenders did not significantly differ in their overall SPS values, an analysis of individual items revealed some notable group differences: AOD problems kept them from meeting responsibilities at work, school, or home (h = -0.29); they repeatedly used AOD when it made the situation unsafe (h = -0.55); and caused them to give up important activities at work, school, or home (h = -0.28). Both regular and ever TS attenders reported more emotional problems and more prior treatment for mental health problems. It may be that emotional problems are more likely to motivate patients with TS experience to turn to treatment. Alternatively, TS experience may be more likely to increase their recognition of their own emotional problems.

### Characteristics of TS experience

It can be informative to study persons who regularly attend TS group meetings immediately prior to applying for professional treatment. Such applicants may have engaged in the TS groups in a way that was not as intense as more stable TS attenders. Since the GAIN includes evaluation of specific TS experiences, we employed this among treatment enrollees who had been regular TS attenders, as illustrated in Table 4. Patients who were regular attenders were active in socializing in the fellowship, as a large majority shared at meetings and felt they were understood by others at meetings. On the other hand, more intensive involvement in the fellowship was less common, as only a minority reported experiencing a spiritual awakening, had a sponsor, served as a sponsor themselves, or considered TS as important in their lives. Further research into distinguishing TS members who are more intensely involved in TS-based recovery from those who are as involved may help in understanding which aspects of the TS experience are most influential in stabilizing recovery, and that the use of the GAIN may be one way to study this. This can be useful in understanding the nature of TS participation, and in treatment planning, as well.

**Table 4:** Regular TS attenders mean self-help involvement scale score and percent endorsing individual items from scale (*n* = 345)

	Mean/%	SD/Count
**Self-Help Involvement Scale**	13.90	5.51
Shared at meeting	89.0%	154
Had a sponsor	40.8%	71
Talked to sponsor at meeting	39.0%	67
Talked w/sponsor or other members outside meeting	67.3%	113
Asked for help	64.3%	108
Read recovery readings	76.3%	129
Actively worked 12 steps	72.6%	122
Prayed for help	75.4%	126
Felt understood by other people at meeting	82.3%	135
Felt you understood other people’s problems at meeting	82.4%	136
Received advice from meeting	86.0%	141
Agreed with advice from meeting	77.1%	128
Member of a home group	21.5%	35
Helped someone from meeting	35.0%	57
Sponsored someone else	8.6%	14
Performed service at meeting	31.1%	52
Participated in group sponsored events	24.4%	40
Had a spiritual awakening	25.6%	42
Considered 12-step an important part of your life	33.7%	55

Future studies are needed that examine the extent to which pre-treatment TS involvement (alone or in combination with prior treatment admission) influences treatment outcome and could serve as a potential marker for problem severity, complexity, and chronicity. Such an identifiable marker at admission could offer guidance on level of care placement decisions and could also identify a subset of people at admission who are in need of enhanced engagement efforts to prevent premature treatment termination and in need of assertive post-treatment monitoring and support (e.g. early and prolonged recovery checkups) to enhance long-term treatment outcomes. Such future studies could also evaluate the extent to which pre-treatment exposure to secular or religious alternatives to TS groups has similar or dissimilar effects compared to TS groups. Knowledge of this points out the potential for mutuality in support between TS members and professional caregivers.

### Limitations

Generalization from findings obtained from the Haymarket Center population to other treatment settings, particularly those outside the United States, has its limitations, as TS experiences can vary relative to the demographics of the local populations and to respective programs’ treatment orientation. Although TS involvement prior to treatment entry was very common, other issues addressed in the GAIN format may be as much, or more, influential in their impact on respondents’ subsequent experience. Additionally, confirmation of substance use before intake was not confirmed in this data set by urinalyses, outside informants, or follow-up during treatment. Relationships between survey items are also correlational, and causality cannot be inferred.

## Conclusion

By employing a structured interview instrument (the GAIN), we were able to characterize SUD patients’ TS experience prior to treatment entry, thereby obtaining findings on TS experience not typically available. This is illustrated by some key clinical findings: Most respondents (53.7%) had attended TS meetings at some point previously, and some (11%) designated themselves as regular TS members. Those with prior TS experience reported more mental health problems and also experienced less SUD intensity, and fewer among them expressed resistance to accepting abstinence as a goal for treatment. Our findings suggest the value of further investigation of how prior TS experience can impact the subsequent course of patients’ treatment and, ultimately, on its outcome. Such findings can be useful in framing clinical interventions early on in treatment. They also illustrate the potential utility of patients’ TS experience over the course of treatment. Further research into the role of TS experience prior to treatment entry merits consideration.

## Data Availability

No datasets were generated or analysed during the current study.
